# Supplementary material for: HIVprotI: an integrated web based platform for prediction and design of HIV proteins inhibitors
Source: J Cheminform. 2018 Mar 9;10:12. doi: 10.1186/s13321-018-0266-y (PMC5845081; doi:10.1186/s13321-018-0266-y)
Supplement: Supplementary file 2 — Additional file 2. Source code of HIVProtI web server. [file 13321_2018_266_MOESM2_ESM.zip › HIVprotI_Source-code/hivprotI/weka-3-6-6/doc/weka/classifiers/IntervalEstimator.html]

IntervalEstimator


---


|  |  |  |
| --- | --- | --- |
| **Overview** \| **Package** \| **Class** \| **Tree** \| **Deprecated** \| **Index** \| **Help** \| **Weka's home** | |  |
| **PREV CLASS**   **NEXT CLASS** | **FRAMES**    **NO FRAMES**     **All Classes** |  |
| SUMMARY: NESTED \| FIELD \| CONSTR \| METHOD | DETAIL: FIELD \| CONSTR \| METHOD |  |


---


## weka.classifiers Interface IntervalEstimator

**All Known Implementing Classes:**: GaussianProcesses

---

``` public interface IntervalEstimator ```

Interface for classifiers that can output confidence intervals

**Version:**
:   $Revision: 1.2 $

**Author:**
:   Kurt Driessens (kurtd@cs.waikato.ac.nz)

---

| **Method Summary** | |
| --- | --- |
| `double[][]` | `predictInterval(Instance inst, double confidenceLevel)`             Returns an N\*2 array, where N is the number of possible classes, that estimate the boundaries for the confidence interval with a confidence level specified by the second parameter. |

**Method Detail**

### predictInterval

```
double[][] predictInterval(Instance inst,
                           double confidenceLevel)
                           throws java.lang.Exception
```

:   Returns an N\*2 array, where N is the number of possible classes, that estimate
    the boundaries for the confidence interval with a confidence level specified by
    the second parameter. Every row of the returned array gives the probability estimates
    for a single class. In the case of numeric predictions, a single confidance interval
    will be returned.

    :   **Parameters:**: `inst` - the instance to make the prediction for.: `confidenceLevel` - the percentage of cases that the interval should cover. **Returns:**: an array of confidance intervals (one for each class) **Throws:**: `java.lang.Exception` - if the intervals can't be computed


---


|  |  |  |
| --- | --- | --- |
| **Overview** \| **Package** \| **Class** \| **Tree** \| **Deprecated** \| **Index** \| **Help** \| **Weka's home** | |  |
| **PREV CLASS**   **NEXT CLASS** | **FRAMES**    **NO FRAMES**     **All Classes** |  |
| SUMMARY: NESTED \| FIELD \| CONSTR \| METHOD | DETAIL: FIELD \| CONSTR \| METHOD |  |


---
